# Laparoscopic Exploration for Penetrating Abdominal Trauma Caused by Deer Antlers: A Case Report

**DOI:** 10.70352/scrj.cr.25-0738

**Published:** 2026-02-28

**Authors:** Atsushi Yoda, Masashi Tsunematsu, Yuka Takehana, Haruki Yamamoto, Naofumi Kobayashi, Yuka Sugiyama, Shingo Nakashima, Hidetoshi Endo, Shunta Ishizaki, Takuo Takehana

**Affiliations:** Department of Digestive Surgery, Saku Central Hospital Advanced Care Center, Saku, Nagano, Japan

**Keywords:** animal-related penetrating trauma, diagnostic laparoscopy, injury

## Abstract

**INTRODUCTION:**

Penetrating abdominal trauma poses a major challenge in trauma surgery, with most cases resulting from knives or firearms. This report describes a rare case caused by deer antlers and highlights the role of diagnostic laparoscopy.

**CASE PRESENTATION:**

A 77-year-old man presented with epigastric and right flank pain after deer antler penetration. On arrival, he was hemodynamically stable but diaphoretic. Physical examination revealed two abdominal wall wounds without obvious contamination. Ultrasonography revealed free fluid in the upper abdomen. Contrast-enhanced CT identified two abdominal wall defects and intraperitoneal fluid but no solid organ injury. Given the risk of occult organ injury and bacterial contamination from the antlers, emergency diagnostic laparoscopy was performed. Laparoscopy revealed approximately 800 mL of hemoperitoneum. Findings suggested that the antler had penetrated the abdominal wall and falciform ligament and reached the left margin of the hepatoduodenal ligament. Peritoneal lavage and subphrenic drain placement were then performed. The operative time was 90 minutes, and no transfusion was required. The patient was discharged on POD 6 without any complications. Retrospective review of preoperative imaging and operative video revealed a small penetrating liver injury that had achieved spontaneous hemostasis and was considered the source of hemoperitoneum.

**CONCLUSIONS:**

Diagnostic laparoscopy is effective and safe for hemodynamically stable penetrating abdominal trauma, including injuries caused by uncommon mechanisms such as animal horns. While it facilitates accurate assessment and lavage, surgeons must remain aware of its limitations, particularly the risk of missed injuries with complex trajectories or spontaneous hemostasis.

## Abbreviation

PAT
: penetrating abdominal trauma

## INTRODUCTION

PAT remains a major challenge in trauma surgery.^1)^ Although most cases are caused by knives or firearms, the optimal management of hemodynamically stable patients remains debated. Advances in imaging have supported selective nonoperative approaches; however, small visceral or mesenteric injuries may be missed, particularly when the wound tract is complex.^2)^ Diagnostic laparoscopy allows direct visualization, enables lavage and hemostasis, and reduces the likelihood of non-therapeutic laparotomy.^3)^ Although animal-related penetrating injuries are extremely rare, they highlight the importance of thorough evaluation for contamination and occult injuries.^4)^ At the same time, such injuries may pose unique diagnostic challenges because of their irregular trajectory and potential involvement of anatomically concealed regions.

We present a case of deer antler-induced PAT successfully managed laparoscopically discussing the indications for laparoscopic exploration in penetrating trauma.

## CASE PRESENTATION

**Fig. 1:**
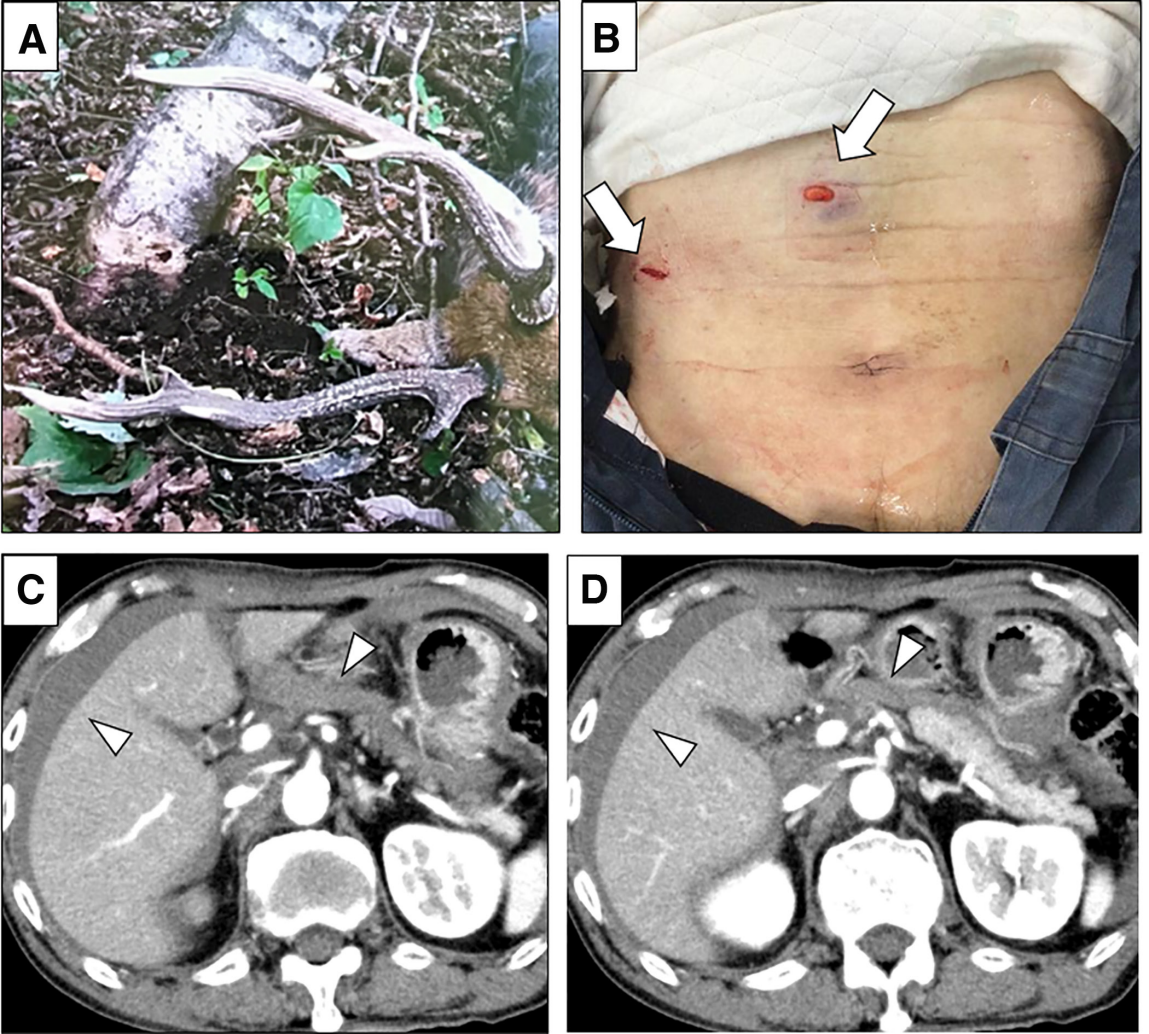
Physical examination findings and preoperative CT. (**A**) The deer antlers that caused the injury were approximately 40 cm in length. (**B**) Two abdominal wall wounds (arrows). (**C** and **D**) Contrast-enhanced CT revealed a large amount of intraperitoneal fluid (arrowheads), with no evidence of active extravasation, free air, or solid organ injury.

A 77-year-old man was injured while attempting to capture a deer caught in a trap. The deer suddenly charged, striking him with its antlers. He was transported to our hospital by emergency services approximately 5 hours after the injury, presenting with epigastric and right flank pain after sustaining abdominal injuries caused by deer antler (**Fig. 1A**). On arrival, his vital signs were stable, although he was diaphoretic. Physical examination revealed two abdominal wall wounds with no obvious contamination: one in the epigastric region and another in the right lateral abdomen (**Fig. 1B**). No injuries were identified outside the abdomen. Ultrasonography revealed free fluid in the upper abdomen. Blood tests showed mild anemia (hemoglobin 12.9 g/dL) and an elevated lactate level (3.03 mmol/L) without metabolic acidosis. Liver enzymes, coagulation parameters, and platelet counts were within normal limits (AST 20 IU/L, ALT 14 IU/L, LDH 163 U/L, peripheral platelet count 24.4 x 10^3^ /μL, and PT-INR 0.92). Contrast-enhanced CT revealed two abdominal wall defects and a large amount of intraperitoneal fluid, with no evidence of active extravasation, free air, or solid organ injury (**Fig. 1C**, **1D**). Given the potential for occult organ injury and bacterial contamination from the deer antlers, emergency diagnostic laparoscopy was performed following tetanus toxoid and 1 g of cefmetazole administration (**Supporting Video**).

**Fig. 2:**
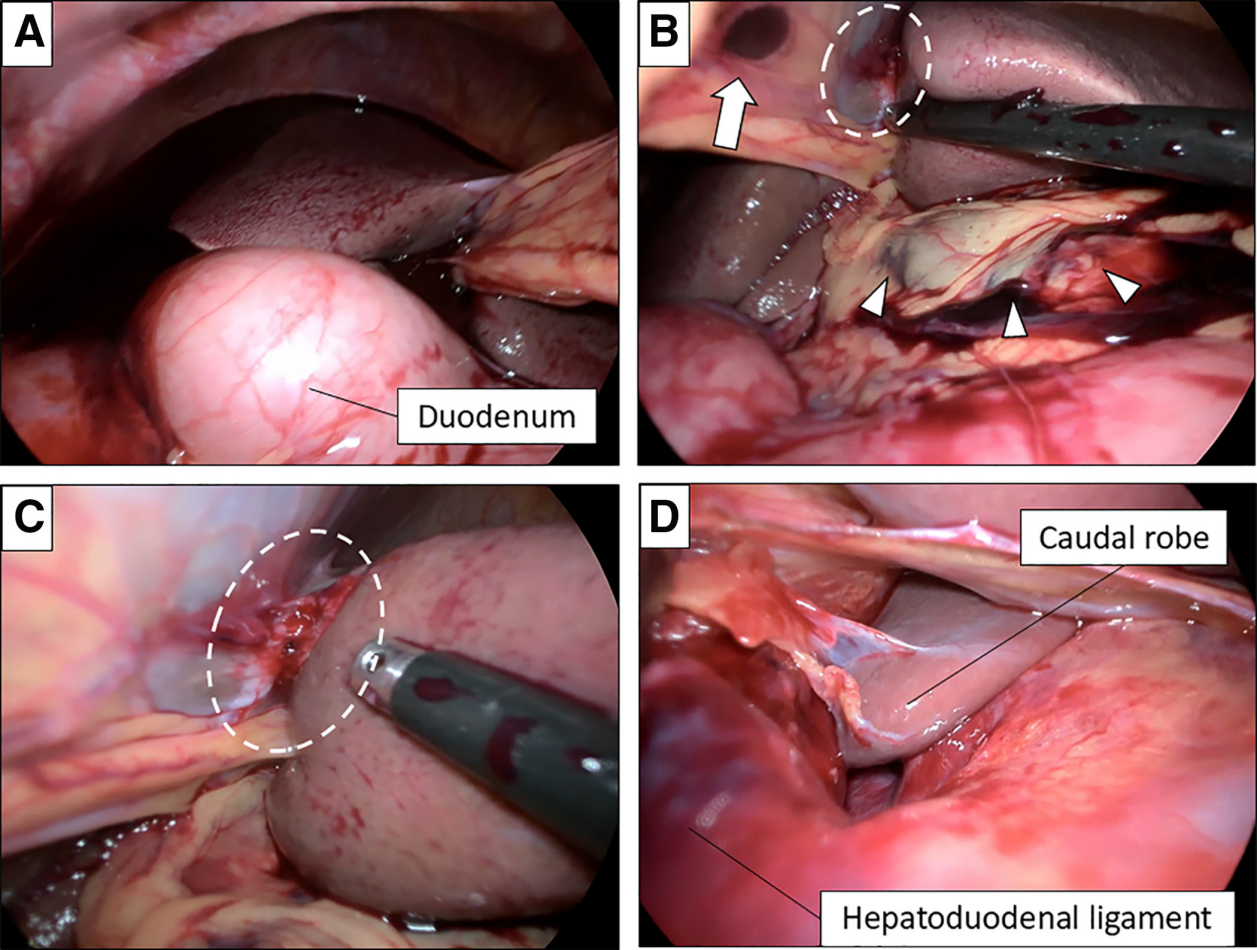
Intraoperative findings. (**A**) Laparoscopy revealed approximately 800 mL of hemoperitoneum (arrow). (**B**–**D**) Injury to the falciform ligament was identified near the epigastric wound (arrow), and the hepatic capsule at the attachment of the round ligament was torn (dashed-line oval), with a hematoma extending along the left margin of the hepatoduodenal ligament (arrowheads).

After pneumoperitoneum (8 mmHg), laparoscopy revealed approximately 800 mL of hemoperitoneum (**Fig. 2A**). Both wounds penetrated the abdominal cavity. Injury to the falciform ligament was identified near the epigastric wound, and the hepatic capsule at the attachment of the round ligament was torn, with a hematoma extending along the left margin of the hepatoduodenal ligament (**Fig. 2B**–**2D**). These findings suggested that the antler had penetrated the abdominal wall and falciform ligament, reaching the left margin of the hepatoduodenal ligament. No active bleeding or additional other organ injuries were observed. After thorough peritoneal lavage with 6000 mL of warm saline, drains were placed in the right and left subphrenic spaces. The operative time was 90 minutes, and no transfusion was required.

Cefmetazole was administered at a daily dose of 3 g through POD 5. The postoperative course was uneventful, and the patient was discharged on POD 6. Bacterial cultures of the ascites obtained intraoperatively were negative. At the 1-month follow-up, the patient exhibited complete wound healing without signs of infection, hernia formation, or functional impairment.

**Fig. 3:**
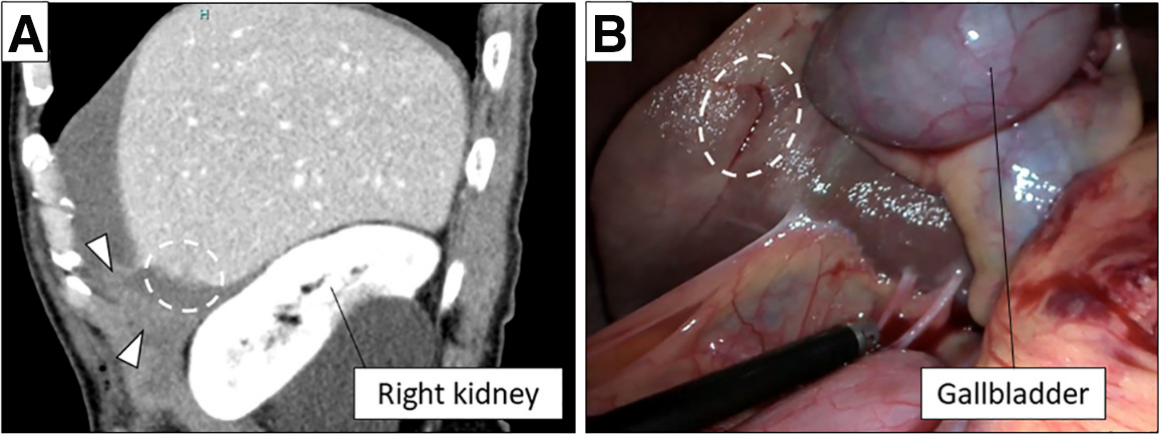
Retrospective review of preoperative CT images and operative video. (**A**, **B**) This review revealed that a large amount of hematoma had accumulated in the right subphrenic space (arrow head), and a small laceration was present on the surface of liver segment 5 (dashed-line oval).

Retrospective review of preoperative CT images and operative video in collaboration with radiologists revealed an overlooked penetrating injury to liver segment 5 (**Fig. 3A**), with an irregular capsular defect consistent with the entry wound in the left lateral abdomen (**Fig. 3B**). This injury was considered the primary source of massive hemoperitoneum.

## DISCUSSION

PAT caused by animals is extremely rare, and deer antler-related injuries are particularly rare.^4)^ Regardless of the mechanism, the key clinical challenge lies in accurately identifying intra-abdominal organ injuries and determining the appropriate surgical approach. Unlike knife or firearm injuries, animal horn-related trauma is characterized by irregular, curved, and contaminated penetrating objects, which can create complex and unpredictable wound trajectories.^5)^ These features increase the risk of occult organ injury and complicate both radiological and surgical assessment.

Hakoda et al. reported a case of bull horn-induced abdominal trauma successfully managed with laparoscopic-assisted hemostasis.^5)^ They emphasized that animal horn injuries often result in atypical penetration pathways that do not correlate with the apparent entry wounds. They noted that posterior organ injuries and tangential parenchymal lacerations may be overlooked if exploration is guided by the presumed trajectory based on skin wounds or initial imaging. The mechanism and clinical challenges described in their report closely resemble those encountered in the present case of deer antler injury.

In modern trauma care, contrast-enhanced CT is central to the initial assessment of hemodynamically stable patients. However, small visceral or mesenteric injuries may be overlooked, particularly when the wound tract is complex.^1)^ Previous studies have reported that up to 20% of hollow viscus injuries in penetrating trauma may be missed on initial imaging.^2,6)^ Animal-related penetrating injuries may further amplify this limitation because the direction, depth, and curvature of horn penetration are often difficult to reconstruct radiologically. Therefore, diagnostic laparoscopy has gained acceptance as an important option for hemodynamically stable patients when imaging findings are inconclusive.^7)^

The present case also highlights an important limitation of diagnostic laparoscopy in the setting of animal-related penetrating trauma. Although approximately 800 mL of hemoperitoneum was identified intraoperatively, no active bleeding source was initially recognized. Retrospective review of preoperative CT images, and intraoperative video with radiologists revealed a liver injury in segment 5. The irregular capsular laceration on the liver surface behind the gallbladder had already achieved spontaneous hemostasis and was therefore not apparent intraoperatively. This overlooked injury likely accounted for the massive hemoperitoneum (approximately 800 mL), given the penetrating trajectory from the right lateral abdominal entry wound toward the anterior liver. This finding underscores that pneumoperitoneum, limited exposure of posterior structures, and spontaneous hemostasis may obscure certain injuries during laparoscopic exploration.

The presence of an overlooked minor hepatic injury does not indicate failure of the laparoscopic approach. The primary goals of trauma surgery—accurate diagnosis, hemostasis, prevention of contamination-related complications, and avoidance of unnecessary laparotomy—were achieved. The patient remained hemodynamically stable, required no transfusion, and experienced an uncomplicated postoperative course.

In addition to mechanical injury, infection risk represents a distinctive concern in animal horn-related penetrating trauma. Although no studies have specifically characterized the microbiological contamination of animal horns, it is reasonable to extrapolate from the literature on animal bite wound infections, which frequently involve polymicrobial flora, including Gram-negative enteric bacteria, anaerobes such as Bacteroides species, and soil-associated organisms including Clostridium species.^8)^ Accordingly, broad-spectrum antibiotic coverage and appropriate tetanus prophylaxis are essential, even in the absence of overt gastrointestinal perforation. In the present case, perioperative antibiotic therapy and tetanus prophylaxis were administered, intraoperative ascitic fluid cultures were negative, and no infectious complications occurred. Although clindamycin may be considered in cases with extensive soil contamination or devitalized tissue to enhance anaerobic coverage, it was not administered in this case because there was no evidence of bowel injury, necrosis, or progressive infection, and cefmetazole was deemed sufficient.

## CONCLUSIONS

In summary, animal horn-related penetrating abdominal trauma poses unique diagnostic and therapeutic challenges due to irregular wound trajectories, contamination, and the risk of occult injury. Diagnostic laparoscopy offers significant advantages in hemodynamically stable patients, including direct visualization, lavage, and avoidance of non-therapeutic laparotomy. However, surgeons must remain aware of its limitations and should be prepared for careful postoperative reassessment when clinical findings and intraoperative observations appear discordant.

## SUPPLEMENTARY MATERIALS

Supplementary video 1 Intraoperative findings during laparoscopic surgery for penetrating abdominal trauma caused by deer antlers.

## DECLARATIONS

### Availability of data and materials

Data sharing is unavailable due to patient privacy concerns, but may be considered by the corresponding author on reasonable request.
